# Normal incidence filters using symmetry-protected modes in dielectric subwavelength gratings

**DOI:** 10.1038/srep36066

**Published:** 2016-11-08

**Authors:** Xuan Cui, Hao Tian, Yan Du, Guang Shi, Zhongxiang Zhou

**Affiliations:** 1 Department of Physics, Harbin Institute of Technology, Harbin 150001, China

## Abstract

We investigate narrowband transmission filters based on subwavelength-grating reflectors at normal incidence. Computational results show that the filtering is realized through symmetry-protected mode coupling. The guided mode resonances introduced by the slab layer allow flexible control of the filter frequencies. The quality factor of the filters could exceed 10^6^. Dielectric gratings can be used over the entire range of electromagnetic waves, owing to their scale-invariant operations. Owing to the high refraction index and low index dispersion of semiconductors in the infrared range, these filters can be applied over a broad range from near infrared to terahertz frequencies.

Optical resonators with high quality-factor (Q) modes play crucial roles in modern photonic technologies, with applications including sensing^1^,^2^, filtering^3^, display technologies^4^, lasers, and optical interconnects^5^. The p lanar design has attracted significant research attention because of benefits such as easy fabrication and its potential for on-chip integration with other optoelectronic components^6^,^7^,^8^. Moreover, dielectric gratings have become attractive planar components for optical engineering, owing to their scale-invariant operation in the visible, near-infrared, mid-infrared, and terahertz spectral regions. Recently a novel subwavelength structure, the high contrast grating (HCG), has been attracting attention^9^,^10^,^11^,^12^. Owing to its remarkable performance in terms of dispersion, reflectivity, and bandwidth^13^, HCGs are widely used in quantum cavities^14^,^15^, vertical-cavity surface-emitting lasers (VCSELs)^16^, polariton lasers^17^, and optomechanical nanoresonators^18^, replacing the conventional distributed Bragg reflector. Furthermore, improved structures termed hybrid gratings (HGs)^19^ or zero-contrast gratings (ZCGs)^20^ have been demonstrated that have solved the discontinuity of structure. These structures have an additional guided layer that induces coupling between guided modes and grating modes, which increases the frequency and angle range of high reflectivity^21^. The coupling of the optical modes of these structures results in intricate transmission properties, including narrowband transmission filtering through symmetry breaking. Based on HCG reflectors, through introducing some symmetry-breaking methods, such as oblique incidence and non-rectangular gratings, the symmetry-protected modes can be coupled to external radiation, resulting in high-Q filtering^22^,^23^,^24^. However, HCG filters have discontinuous structures and cannot easily control the filter frequencies because the grating modes influence not only the filter frequencies but also the high reflection bands.

In this letter, we utilize the coupling between guided modes and radiation modes via breaking the symmetry of the zero-contrast gratings, and thus realizing narrowband transmission filters. We investigate the effect of the different modes in our structure. The TM1 guided-modes-coupling facilitates simple control of the filtered frequency by changing the thickness of the slab layer. Meanwhile, the strength of the coupling, as well as the quality factor, is determined by the symmetry-breaking level. Moreover, these filters can be utilized in the spectral range from the near-infrared to terahertz regions.

## Results

**Figure 1:**
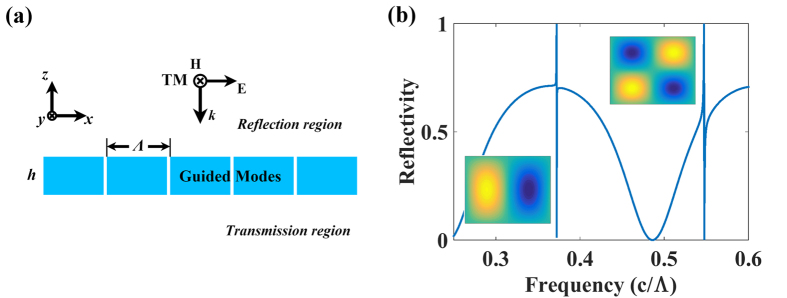
(**a**) Grating schematic with the dimensions, incident, and transmitted fields defined. The grating dimensions include the period (Λ), height (*h*_1_), and duty cycle (*η*), which is defined as the ratio of the high permittivity (*ε*_d_) region (*L*) to the grating period. The incident light is transverse magnetic (TM) polarization defined with the magnetic field directed in the y-direction. (**b**) Reflectivity as a function of frequency (c/Λ) with the parameters *h* = 0.6, *η* = 0.99, and *ε*_d_ = 11.9. Insets in (**b**) illustrate the magnetic field profiles (*H*_y_) corresponding to the Fano resonances TM_1_ and TM_2_.

A uniform dielectric slab possesses guided modes with infinite lifetime. When a periodic structure is introduced into a dielectric slab, such as air holes or slits, it becomes a photonic crystal slab in which some guided modes can couple to radiation modes and possess a finite lifetime. These modes therefore become guided resonances. They are termed “guided” because they are closely related to the guided modes in a uniform slab^25^. The presence of a guided resonance in a slab is manifested as a Fano line shape superimposed on an otherwise smooth background in the transmission spectrum^26^. Figure 1a shows a schematic of a slab with periodic slits, and defines the dimensions and incident and transmitted fields. The grating dimensions include the period (Λ), height (*h*_1_), and duty cycle (*η*), which is defined as the ratio of the high permittivity region (*L*) to the grating period. The frequency (*f*) is normalized by (c/Λ) in which c is the speed of light in vacuum (in the calculations, we set light speed to unity). In this letter, we utilize transverse magnetic (TM) polarization, which is defined with the magnetic field directed in the *y*-direction. The light is incident from the *z*-direction. The dielectric permittivity (*ε*_*r*_) is set to 11.9, which is a typical value for silicon in the infrared and terahertz range, and the air permittivity is unity.

The results of the reflectivity and magnetic field profile (*H*_y_) shown in Fig. 1b were computed with the finite element method (FEM), using the COMSOL Multiphysics software package, with the parameters *h*_1_ = 0.6 and *η* = 0.99 at normal incidence. In the figure, two Fano resonances (*f* = 0.372 and 0.548) can be seen to superimpose upon the classical Fabry-Perot transmission background. The magnetic field profiles (*H*_y_) are shown at resonance frequencies to illustrate the guided resonances. The presence of air slits in the slab lowers the translational symmetry of the structure from continuous to discrete symmetry, and thereby some guided modes can couple to radiation modes^25^. The Fabry-Perot transmission background has not been significantly modulated by these resonances because the slits are narrow.

**Figure 2:**
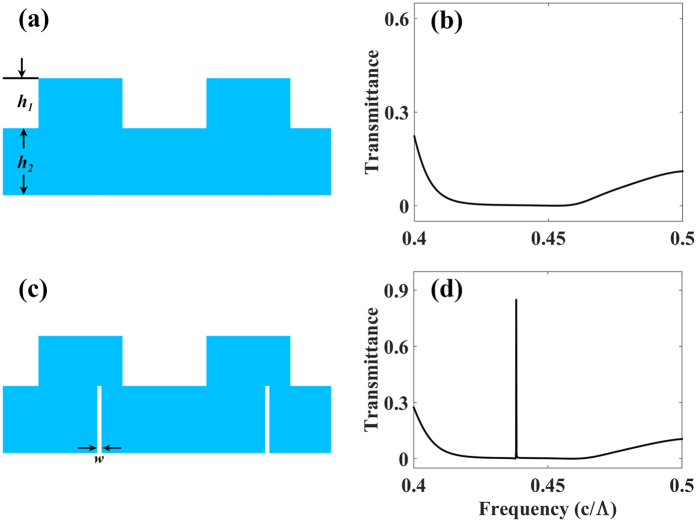
Grating cross sections (**a**,**c**) with corresponding normal incidence response (**b**,**d**). Geometries include (**a**) ZCG reflectors, (**c**) ZCGs with slits in asymmetric position, with the parameters: *η* = 0.5, *h*_1_ = 0.685 Λ, and *h*_2_ = 0.37 Λ. The slit width(*w*) is set to 0.02 Λ. The blue areas and white areas in (**a**,**c**) represent the high index material and air respectively.

In an analogy to the translational-symmetry breaking in photonic crystal slabs, the internal modes of a ZCG could couple to radiation modes through the mirror symmetry breaking. ZCG reflectors have a broadband opaque background, and the high reflection range can be optimized by modifying the slab waveguide layer (*h*_2_) below the grating. We consider two cross-sectional grating geometries, the ZCG cross section previously optimized to act as a broadband reflector and periodic slits in the slab layer, as shown respectively in Fig. 2a,c. In Fig. 2c, the slits are etched in the asymmetric position in the slab layer. The corresponding transmittances at normal incidence with a TM polarization are shown in Fig. 2b,d, with the parameters *η* = 0.5, *h*_1_ = 0.685 Λ, and *h*_2_ = 0.37 Λ. The slit width (*w*) is set to 0.02 Λ. As shown in Fig. 2a, a ZCG reflector is opaque over a wide band of wavelengths. In Fig. 2c, when the slits are asymmetrically etched, the mirror symmetry of the structure is broken, resulting in the narrow transmission band filtering.

**Figure 3:**
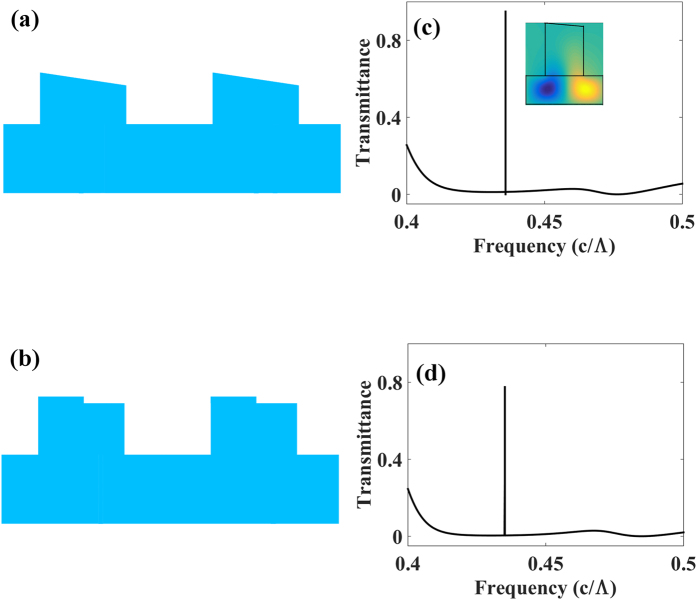
Grating cross sections (**a**,**b**) with corresponding normal incidence response (**c**,**d**) and the magnetic field profiles (*H*_*z*_) at resonance frequency. Geometries include (**a**) right trapezoid (**b**) asymmetric groove.

In ZCG reflectors, the broadband of high reflectivity is a result of the coupling between guided modes (magnetic fields are confined in the slab layer), waveguide array (WGA) modes (magnetic fields are confined in the grating bars), and Fabry-Perot modes^22^. The resonance frequencies, namely the filter frequencies, are determined by the internal mode resonances, including the WGA modes and guided modes. Figure 3 shows another two asymmetric grating structures (right trapezoid and asymmetric groove) and their corresponding transmission, showing that the filter frequencies are irrelevant to how the grating symmetry is broken. The right trapezoid, asymmetric slits, and groove structure have almost the same resonance frequencies for the given structure parameters.

The high reflectivity of a traditional HCG is purely a result of WGA mode coupling. The coupling of WGA modes is sensitive to the phase changes of the interface; the alteration of the HCG structure might greatly change the reflection properties. Therefore, it is difficult to control the spectrum properties of HCG based filters. ZCG reflectors refer precisely to the same interface without ambiguity; thereby eliminating local interface reflections and phase changes. The coupling between guided modes and WGA modes provides the flexibility to control the transmittance of the structure. The magnetic field profiles (*H*_*z*_) of a right trapezoidal structure at the resonant frequency, illustrated in Fig. 3c, show the resonance of the guided modes. The coupling of guided modes provides the possibility to control the filtered frequencies.

**Figure 4:**
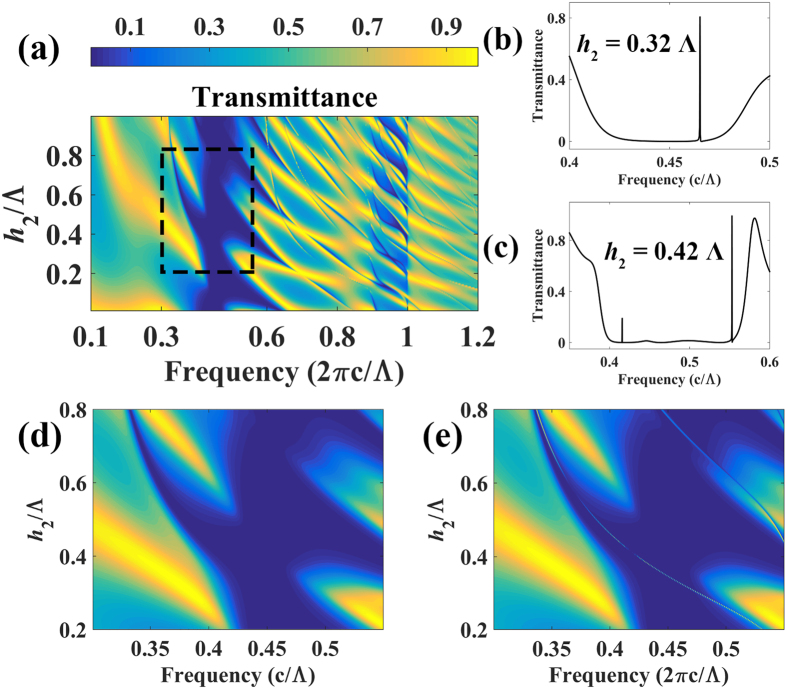
(**a**) Transmittance contour map as a function of frequency and slab-layer thickness with *η* = 0.5, *h*_1_ = 0.685 Λ and no slits. Transmittance contour map using the same parameters with (**e**) and without (**d**) asymmetric slits. The transmittance in (**b**,**c**) represent the slab thickness *h*_2_ = 0.32 Λ and *h*_2_ = 0.42 Λ.

As mentioned above, the resonance frequencies are determined by the internal modes of a ZCG. The transmittance contour map of a ZCG, illustrated in Fig. 4a, shows how the internal modes influence the transmission versus the normalized frequency (c/Λ) and slab-layer thickness *h*_2_ for a surface-normal incident TM-plane wave, grating-layer thickness *h*_1_ = 0.685 Λ, and no slits. As shown in the contour map, when the slab layer is thin (in the bottom area), an opaque region exists owing to the coupling of WGA (grating) modes. As the slab-layer thickness increases, guided modes emerge which then couple with WGA modes and expand the high-reflectivity range. The effect of the thickness of the slab layer provides the possibility to control the filter frequencies, analogous to that in a slab waveguide. Taking the asymmetric slits structure for example, the contour map, shown in Fig. 4e, clearly illustrates the relation between the filter frequencies and the slab-layer thickness. The transmittance with the same parameters but with no slits is marked in Fig. 4a with a dashed line, and enlarged in Fig. 4d in detail. The light line in Fig. 4e, illustrates that the dependence of the filter frequency on the slab layer thickness resembles the dispersion relationship of guided modes. The thickness of the slab layer affects the phase-matching condition of the guided modes, thus determining the filter frequencies. Figure 4b,c show the spectrum with different *h*_2_. As the slab layer thickness increases, different types of modes and higher-order mode resonances emerge (details in SI).

**Figure 5:**
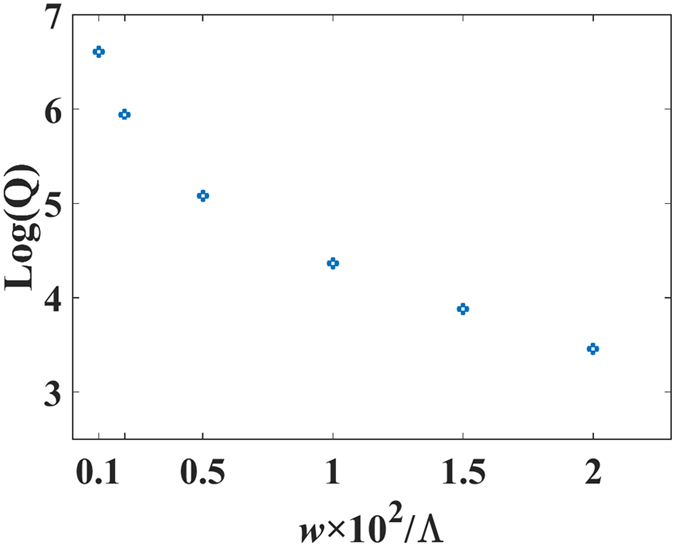
Filtering quality factor (Q) vs. the slit width.

The quality factor (Q) is mainly determined by the strength of the coupling, which depends on the etching angle of the right trapezoidal structure or the slit width of the asymmetric slit cross section. Figure 5 illustrates the influence of the slit width (*w*) on the quality factor, with the parameters *h*_2_ = 0.37 Λ. A similar response is exhibited by the other slab thicknesses and trapezoidal gratings. It is demonstrated that the quality factor has a negative correlation with the slit width and can exceed a value of 10^6^ at *w* = 0.001 Λ. In theory, the quality factor can be infinite as the slit width or etching angle approaches zero. However, optical absorption and experimental constraints limit the attainable Q in practice. Because the resonance frequencies are determined by grating parameters, fabrication errors in the main gratings, including slab layer thickness and duty cycle, extend the full-width-at-half-maximum (FWHM) bandwidths of the resonances and thus reduce the quality factor. Similarly, trapezoidal gratings have a high Q (>10^6^) when the etching angle is less than 10°. Meanwhile, the resonance frequencies are minimally affected by the etching angle (detailed discussion in SI). Therefore, the fabrication accuracy of etching angle has little influence on the quality factor.

Here, the permittivity of the grating is set to 11.9, which is a typical value of silicon in the infrared range. Silicon and many other semiconductor materials have a high refractive index (2.8–3.5) and little dispersion from the near-infrared to the terahertz region, which enables simple application of the filters in the infrared and terahertz region through modification of the grating parameters. All simulations are performed with normalized units. Therefore, the structure can be easily designed by adjusting Λ for a certain frequency. For example, for the traditional optical communication region, we set the resonance wavelength to 1.55 μm using *h*_2_ = 0.37 Λ with the structure in Fig. 3a, and the opaque range from 1.44 μm to 1.66 μm with the following structure parameters: Λ = 0.683 μm, *h*_1_ = 0.468 μm, and *h*_2_ = 0.308 μm. For the infrared use, this dimension of blazed gratings could be fabricated using advanced nanofabrication techniques^27^,^28^, and micron-scale slits are easily fabricated for use in the terahertz range. The large real and imaginary part of the permittivity of metal in the terahertz region is the primary barrier, causing high losses and the inability to support surface modes. However, semiconductor materials, which could be treated as dielectrics, can directly apply the optical theory in the terahertz region. Moreover, the fabrication techniques for semiconductor materials are mature. This dielectric structure based on semiconductor materials is a promising solution for terahertz devices. Therefore, we can modify the Fano resonance at 1 THz and the opaque range from 0.93 to 1.04 THz by adjusting the parameters to Λ = 132 μm, *h*_1_ = 91 μm, and *h*_2_ = 60 μm.

## Discussion

In conclusion, we proposed a subwavelength-grating-based filter at normal incidence. By breaking the symmetry of the grating, coupling between radiation modes and guided modes, supported by the slab layer, yields narrow transmission bands within an opaque background. Owing to the coupling of guided modes and WGA modes in the ZCG reflector, the spectrum properties can be controlled separately by both modes. In our structure, the filtering ability results from guided resonance coupling, which could be controlled by the thickness of slab layer, thus leading to flexibility of the design of the filter frequencies. Despite the material and fabrication limitations, the quality factor of the filters can reach more than 10^6^. In addition, owing to their scale-invariant operation, these dielectric gratings have promising applications as planar components for optical engineering in the wavelength range from the visible to terahertz regions.

## Methods

The simulations are conducted with a commercial finite-element method (FEM) package COMSOL Multiphysics. 2-D simulation area is established using wave optic module with periodic boundary and port excitation. Perfectly matched layers are added to simulate free space. The x and y directions and wave polarization are defined in Fig. 1a. The dielectric permittivity (*ε*_*r*_) is set to 11.9, and the air permittivity is unity. The length unit is normalized by the period (Λ), and the frequency (*f* ) is normalized by (c/Λ).

## Additional Information

**How to cite this article**: Cui, X. *et al.* Normal incidence filters using symmetry-protected modes in dielectric subwavelength gratings. *Sci. Rep.* **6**, 36066; doi: 10.1038/srep36066 (2016).

**Publisher’s note:** Springer Nature remains neutral with regard to jurisdictional claims in published maps and institutional affiliations.

## Supplementary Material

Supplementary Information
