# Supplementary material for: Normal incidence filters using symmetry-protected modes in dielectric subwavelength gratings
Source: Sci Rep. 2016 Nov 8;6:36066. doi: 10.1038/srep36066 (PMC5099914; doi:10.1038/srep36066)
Supplement: Supplementary Information [file srep36066-s1.pdf]

Supporting Information for:

Normal incidence filters using symmetry-protected modes in dielectric subwavelength gratings

Xuan Cui,[1] Hao Tian,[1,*] Yan Du,[1] Guang Shi[1] and Zhongxiang Zhou,[1]

[1] Department of Physics, Harbin Institute of Technology, Harbin 150001, China

tianhao@hit.edu.cn

- **The difference between WGA modes and guided modes in subwavelength gratings**

There are two main optical modes in the subwavelength gratings, waveguide-array modes(WGA) and guided modes(GM). WGA modes are coupled in the grating bars, which play a key role in the transmission properties of high-contrast gratings. Through the symmetry-breaking of the gratings, these two kinds of modes can be coupled to radiation modes, realizing Fano-type resonances. The magnetic field profiles (H_y), illustrated in Figure 1S, show their difference. The resonance at $f = 0.41$ is a result of coupling of guided modes, where the magnetic fields are confined in the slab waveguide layer. On the other hand, the coupling of WGA modes ($f = 0.55$) is due to the phase match in the grating bars. Therefore, as show in Figure 2S, the change of the thickness of the slab layer mainly affects the coupling of guided modes. However, the WGA modes is barely influenced by the thickness of slab layer.

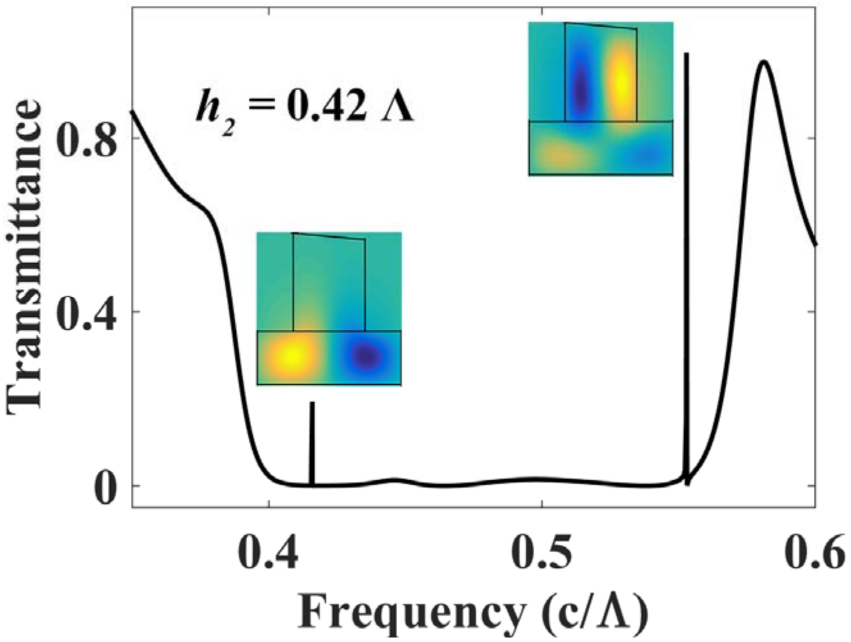

Figure 1S. The transmittance with the slab thickness: h_2 = 0.42 Λ and the magnetic field profiles (H_y) corresponding to the TM_1 guided mode and TM_1 WGA mode.

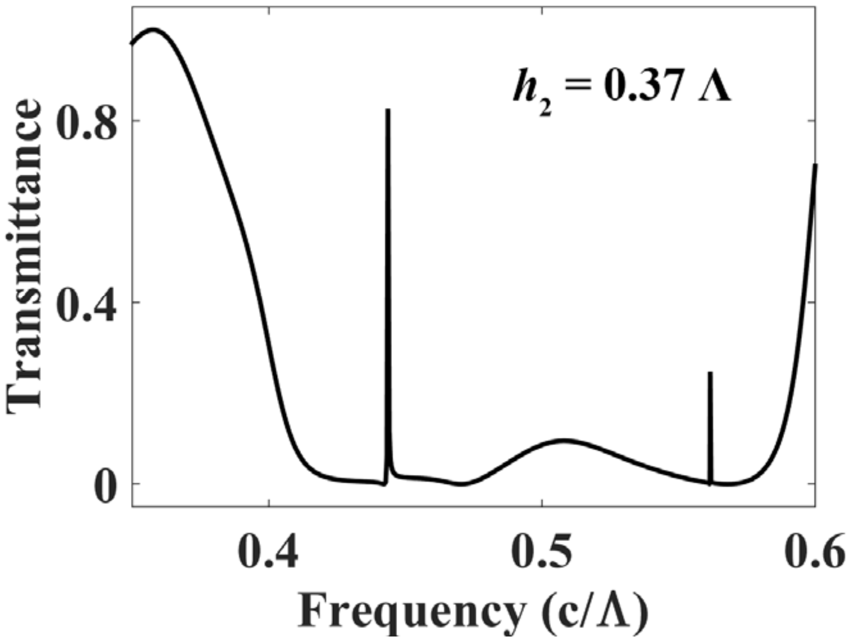

Figure 2S. The transmittance with the slab thickness h_2 = 0.37 Λ.

- **The limitation of quality factor**

There are two main limitation of the filtering quality factor: imaginary part of material's refractive index and fabrication accuracy. In our simulation the material's permittivity is set as 11.9 and lossless. It is an approximate value for silicon in the infrared range and terahertz range. In fact, the silicon has absorption in these range. The following figure shows the absorption versus wavelength for high purity Si at different temperatures. Temperatures are 1. 300 K; 2. 473 K; 3. 573 K; 4. 623 K; 5. 673 K. (Runyan, W. R., *Technology Semiconductor Silicon,* McGraw-Hill Book Company, 1966)

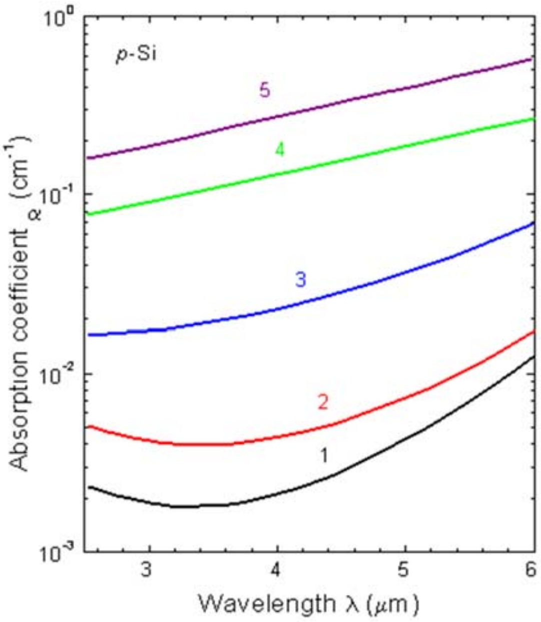

Figure 3S. the absorption versus wavelength for high purity Si at different temperatures. Temperatures are 1. 300 K; 2. 473 K; 3. 573 K; 4. 623 K; 5. 673 K.

we can see the purity silicon has less than 10^{-2} cm^{-1} absorption in infrared range at room temperature. And the absorption of n-Si has the same level. We use the equation $\alpha = 2\omega k/c$, where k is the imaginary part of refractive index. We derive the k is about 10^{-6}i. The transmittance of the 8 degree right trapezoidal structure using the lossy material is showed in the following Fig. 4S(a), and the same structure with the lossless material is showed in Fig. 4S(b).

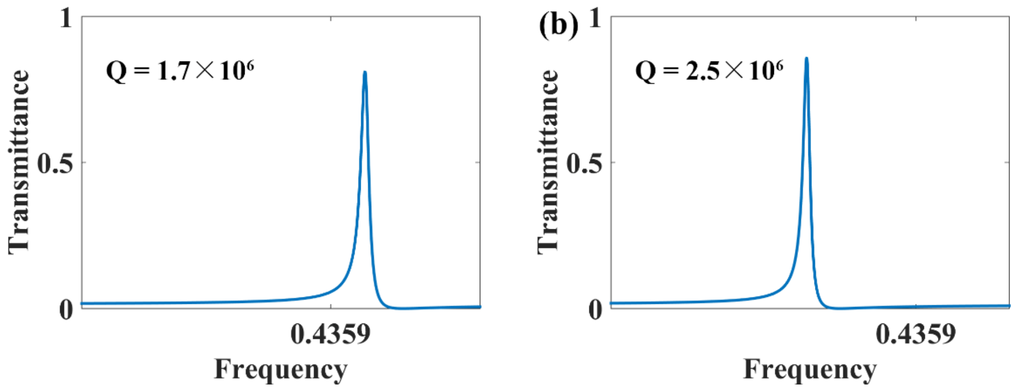

Figure 4S. The transmittance with (a) lossy silicon and (b) loss free silicon model.

the results show that the quality factor is reduced to 1.7×10^6.

Second limitation is the fabrication accuracy. In our structure the filtering ability is result from the coupling between the guided modes resonances and the radiation modes through the symmetry breaking. Therefore, how the symmetry is broken has little influence on the filtering ability. From the main text, we can see that the asymmetric slits, trapezoid gratings and asymmetric groove have the same effects. Thus we can choose different methods to break the symmetry using in different wavelength range. For example, the blazed gratings are routinely fabricated in the infrared dimension; and for the terahertz range the micron scale slits are easy to etched.

Furthermore, owing to the guided resonance is determined by the grating structure the accuracy of the fine fabrication, such as the accuracy of the etched angle, does not affect the quality factor. The following figure shows the filter frequency against the etched angle in the right trapezoid structure. It shows that the perturbation of the etched angle(θ) barely affects the resonance frequencies (f). Around 10°, the influence is about: $\frac{\partial f}{\partial \theta}\approx 3.2\times10^{-6}$. Therefore, the quality factor of the filters can reach 10^6.

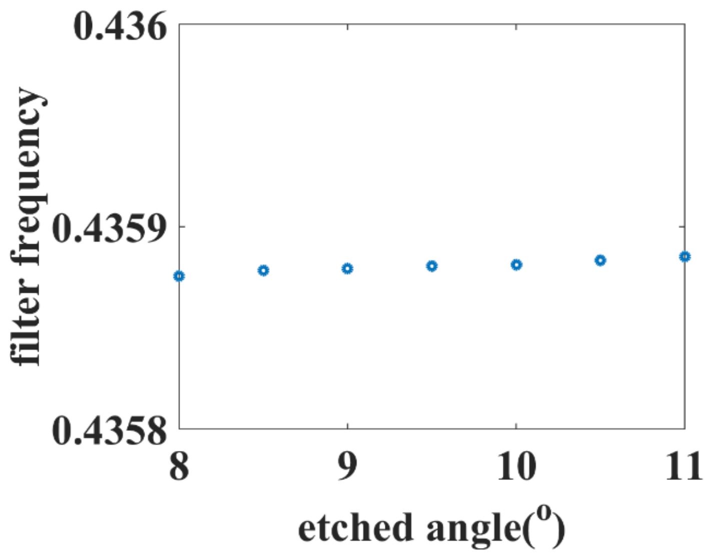

Figure 5S. Filter frequency against the etched angle in the right trapezoid structure
